# Telehealth: Acceptability, clinical interventions and quality of life in peritoneal dialysis

**DOI:** 10.1177/2050312116670188

**Published:** 2016-09-30

**Authors:** Vishal Dey, Audrey Jones, Elaine M Spalding

**Affiliations:** Renal Unit, University Hospital Crosshouse, Kilmarnock, UK

**Keywords:** Peritoneal dialysis, quality of life, telehealth

## Abstract

**Introduction:**

Telehealth technologies are being widely adopted across the globe for management of long-term conditions. There are limited data on its use, effectiveness and patient experience in end-stage renal disease. The aim of this pilot project was to explore patient acceptability of technology and evaluate its effect on clinical interventions and quality of life in patients undergoing peritoneal dialysis.

**Methods:**

Peritoneal dialysis patients were provided with computer tablets (PODs). PODs contained a knowledge database with treatment- and symptom-based questionnaires that generated alerts for the clinical team. Alerts were reviewed daily and followed up by a telephone call or clinic visit. Interventions were at the discretion of clinicians. Data were recorded prospectively and quality of life and Quebec User Evaluation of Satisfaction with assistive Technology questionnaires evaluated at the start and end of the programme.

**Results:**

In all, 22 patients have participated over 15 months. The mean age was 61.6 years and PODs were utilised for an average of 341.9 days with 59.1% choosing to continue beyond the study period. We received a total of 1195 alerts with an average of 2.6 alerts per day. A total of 36 admissions were avoided and patients supported to self-manage on 154 occasions. Quebec User Evaluation of Satisfaction with assistive Technology scores remained high throughout the programme although no improvement in quality of life was seen.

**Discussion:**

Telehealth is useful to monitor patients with renal failure on peritoneal dialysis. It is acceptable across age groups and provides an additional resource for patients to self-manage. Satisfaction scores and retention rates suggest a high level of acceptability.

## Introduction

The number of people living with significant long-term conditions is rising in an era of improved healthcare and an ageing population. It is estimated that the total number of people with at least one chronic condition will rise to 18 million in the United Kingdom by 2025.^1^ Recent data from Quality and Outcomes Framework Disease Register comparing 2006–2007 to 2010–2011 data shows a 45% increase in chronic kidney disease (CKD) only second to cancers (79% change).^2^ With long-term conditions forming 70% of the total healthcare budget in England and many patients wishing to gain better understanding and avoid admissions,^3,4^ new initiatives are being constantly explored. A recent report from a survey of 2500 people by the Institute of Public Policy research, a UK’s leading progressive think tank, found only 20% of people had used assistive technologies to monitor their health and maintain independence.^4^ The use of telecare or telehealth medicine to support self-management has shown promising results, but most of the studies have been limited to chronic obstructive pulmonary disease (COPD), heart failure and diabetes. Data on the use of this technology in people with other complex conditions such as end-stage renal disease (ESRD) are sparse.

NHS Ayrshire & Arran (A&A) provides peritoneal dialysis (PD) services to the highest number of prevalent patients with ESRD on PD in Scotland (n = 37, 10% of total patients on renal replacement treatment at A&A, 31 December 2014).^5^ The geography of the area dictates that people living in remote areas have to travel significant distances to access specialist medical care that is both expensive and time-consuming (up to 2.5 h one-way journey). For those requiring renal replacement therapy (RRT), PD allows the freedom to carry out treatment at home. In the event of illness or machine malfunction, the default position historically was for patients to attend the parent unit at the main hospital at the earliest opportunity. This necessarily resulted in time and cost implications for patients and staff alike.

We set up a pilot project using telehealth technology with the belief that it would help improve patient experience, promote self-management skills and allow early recognition and resolution of medical problems without the need for patients to attend the parent unit.

## Methods

All adults >18 years of age with ESRD on PD were invited to participate. Subjects were recruited after receiving information about the project following focus group meetings or during routine clinic visits. There were no exclusion criteria apart from those not willing to participate.

### Telemedicine system in PD patients

**Figure 1.**
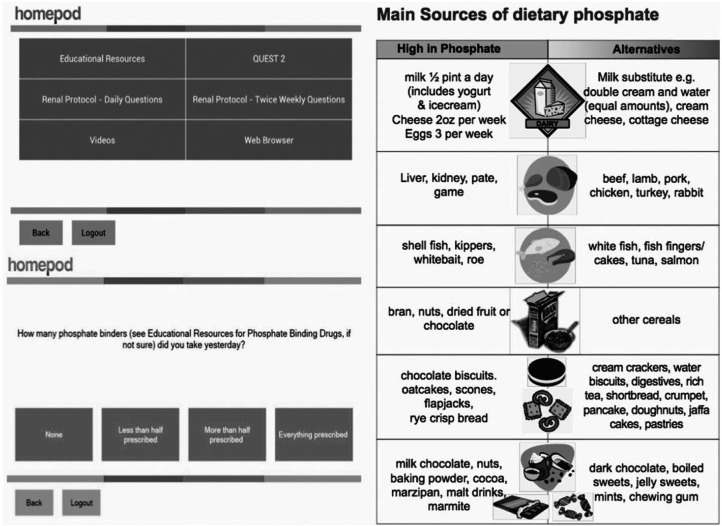
Sample screens of the application.

The application consisted of specialised software that was developed by the clinical team and uploaded into PODs. Each patient was given a POD, weighing scales and blood pressure machines that integrated with the software via Bluetooth technology. Patients were asked to record their vital data (weight, blood pressure, dialysis exchanges and ultrafiltration volumes) and answer a set of questions (worsening swelling, shortness of breath, fever, abdominal pain, tenderness around PD catheter site and alarms from PD machine) on a regular basis. A twice-weekly questionnaire to assess dietary habits and medication intake patterns, and diet sheets along with web links formed part of the knowledge database in the PODs. Patients were able to access their own medical information via ‘Renal Patient View’ a nationally developed web portal. No data collection occurred during hospital admissions. Sample screens of the application are shown in Figure 1.

The application allowed remote monitoring of treatment regimes; early recognition of problems related to dialysis and promoted self-management skills. The PODs were not intended to be an emergency medical device and patients were advised to contact the medical team in the usual manner for urgent or immediate medical attention.

### Data transmission

Data were transmitted from the PODs with a roaming Subscriber Identity Module (SIM) card in an encrypted form. To ensure adequate reception, a site engineer installed PODs at patients’ residences. Patients were provided a 15- to 30-min one-to-one training and free phone number for technical issues. There were no associated costs to the patient apart from the ability to charge the device from power points. All devices were protected by passwords, and no patient identifiable information was stored within the devices. Clinicians were able to send non-urgent single or group messages to patients via the PODs.

### The clinical user interface

Alerts were regularly reviewed by the nursing team using a clinical user interface (CUI; a desktop application, later developed into a secure web portal) and followed up by a telephone call, home or clinic visit as deemed necessary. Vital data that fell outside individually predefined values were highlighted as red alerts. Alerts that needed dietetic or pharmacy input (amber alerts) were passed to colleagues via email or telephone calls. Patients were followed up directly by the relevant department. The CUI was installed at multiple computers across renal services, and nurses, dieticians, pharmacists and doctors received training prior to its use. Data on all actions were recorded prospectively and reports generated for each event until the problem was resolved.

## Outcomes measurement

### Clinical interventions

Clinical specialist nurses, dieticians and pharmacists collected data on interventions, prospectively from alerts generated following patient review. These were categorised into avoidance of admission, support to self-manage, dietary advice, telephone calls, home visits and clinic visits. Clinical interventions were facilitated by examination of POD data related to blood pressure trends, weight gain, pedal oedema or worsening shortness of breath. Interventions to self-manage at home included the following: (1) recognition of fluid-related problems with ability to alter dialysis regimes or perform an extra manual exchange, (2) dietary advice on phone supported by supplementary written material on the PODs, (3) attainment of better understanding of ESRD/PD via web resources and (4) option to access personal electronic clinical records.

### Kidney Disease Quality of Life-36^TM^ and Quebec User Evaluation of Satisfaction with assistive Technology

Kidney Disease Quality of Life (KDQOL-36) English (UK), Version 1.2^6^ and Quebec User Evaluation of Satisfaction with assistive Technology (QUEST) Version 2^7^ were used to assess quality of life (QOL) and user acceptability of technology, respectively. The KDQOL-36 is a self-reported patient survey that includes 36 items (items 1–12, the short form (SF)-12 as generic core; items 13–26, the burden of kidney disease; items 17–28, symptoms/problems and items 29–36, effects of kidney disease). Measurements with higher scores indicate better QOL (range from 0 to 100).^6^

QUEST uses a scoring system based on a scale of 1–5 with 1 indicating *not satisfied at all* and 5 being *very satisfied*. Twelve items, subdivided into three categories, were assessed. These include the assistive device (dimensions, ease in adjusting, safety and security, durability, ease, comfort, effectiveness), services (service delivery, repairs and servicing, professional services, follow-up services) and three most important items.^7^ Both these questionnaires were administered at the start and end of the programme. Standard routine care remained unchanged until problems were flagged by the PODs’ system, at which point clinical interventions were at the discretion of the multi-disciplinary team.

## Ethical reviews and permissions

Written permission was obtained from all patients prior to use of devices. A&A telehealth services obtained appropriate approvals from Information Technology Governance for secure data management. Institute for Matching Person and Technology provided permission for use of QUEST. The medical device (POD) was registered as a class I device (medical device software for telehealth loaded onto third part hardware) with Medicines and Healthcare products Agency Regulatory (MHRA) and ‘CE’ marked as per European legislation. The project was not subject to ethical reviews as this was part of a quality improvement programme.

## Results

### Patient characteristics

A total of 22 patients (10 females and 12 males) on automated PD (APD), all self-caring and not on assisted PD, were identified at the start of the study. The average age was 61.6 years at inception (range 26.4–93.4 years, median 60.3 years). Primary renal diagnosis (PRD) was obtained from the European Renal Association–European Dialysis and Transplant Association (ERA-EDTA) coding system using the related web-based PRD search.^8^ They were grouped as diabetes, familial/hereditary nephropathies, glomerular disease, systemic disease, tubulointerstitial disease and miscellaneous. The average RRT vintage was over 3 years (Table 1).

**Table 1.** Patient characteristics at recruitment.

Females, n (%)	10 (45)
Age in years, average (range)	61.6 (26.4–93.4)
Primary renal diagnosis, n (%)	
Diabetes	4 (18.2)
Familial/hereditary	2 (9.1)
Glomerular disease	5 (22.7)
Miscellaneous	1 (4.5)
Systemic	7 (31.8)
Tubulointerstitial disease	3 (13.6)
PD vintage (months), mean (range)	32.7 (0–139.8)
RRT vintage (months), mean (range)	39.7 (0–194.5)
Days with PODs, mean (range)	341.9 (8.0–458.0)

PD: peritoneal dialysis; RRT: renal replacement therapy.

### Data from the PODs

**Figure 2.**
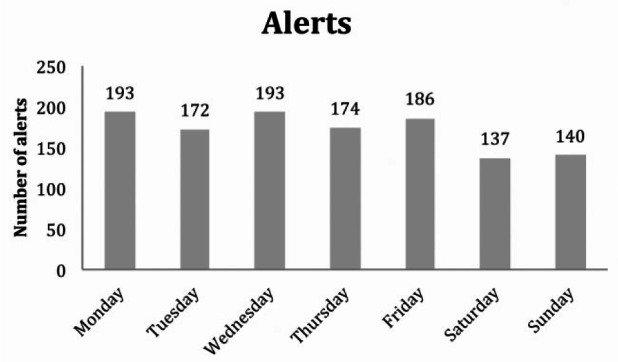
Alerts by day of the week.

We received a total of 1195 alerts, 1074 red, directly affecting clinical care, and 121 amber, related to diet and medication. The distribution of alerts by day of the week is illustrated in Figure 2. In all, 562 alerts (47% of total alerts) lead to an intervention while the remaining 53% required no further action. These alerts were generated mainly for safety reasons, examples of which were single readings of abnormal weight or blood pressure. Further development in the software will help minimise some of these extra alerts.

### Clinical interventions

Data from interventions (Table 3) generated by alerts suggest that telehealth was effective in reducing admissions and supporting patients to self-manage from the comfort of their home. The most frequent reason for admission avoidance was by early recognition of fluid overload. The data about admission avoidance were based on information collected from PODs and the expertise of the of the clinical nurse specialist. A standardised protocol was agreed where patients who demonstrated a persistent increase in weight (more than 1.5 kg over 72 h), had a persistent rise in blood pressure of >20 mmHg (systolic or diastolic) or reported symptoms of worsening shortness of breath or pedal oedema received a telephone call. A judgment for further review, intervention or admission was made using the above information in conjunction with the individual’s other medical co-morbidities (heart failure, significant valvular disease or tendency to fluid overload).

### QUEST and QOL outcomes

Table 2 illustrates the QUEST and QOL scores at the start and end of the programme. QUEST satisfaction scores remained high although there was no significant change in the QOL scores at the end of the programme. The three most important features about the PODs for patients were ease of use, effectiveness and safety.

**Table 2.** Clinical interventions from generated alerts.

Interventions	n
Admissions avoided	36
Medication change	4
Self-manage at home	154
Telephone calls to patients	258
Visits	
General practitioner	8
Home	62
Outpatient PD clinic	15
PD day unit	7
Referrals/advice	
Dietician	16
Pharmacy	2

n: number of patients; PD: peritoneal dialysis.

**Table 3.** QUEST and QOL scores at the start and end of programme.

	Initial	Final	p-Values
QUEST,^a^ mean (SD)			
Device	4.5 (0.5)	4.5 (0.6)	
Service	4.2 (0.9)	4.1 (1.0)	
Total (device + service)	4.4 (0.5)	4.3 (0.6)	
QOL, mean (SD)^b^			
Symptom/problem list	70.2 (17.0)	64.7 (15.8)	0.27
Effects of kidney disease	75.6 (21.5)	69.6 (24.3)	0.37
Burden of kidney disease	55.1 (33.9)	40.9 (24.7)	0.49
SF-12 Physical Health Composite	29.7 (6.1)	31.5 (8.8)	0.54
SF-12 Mental Health Composite	46.2 (10.4)	43.6 (11.6)	0.43

QUEST: Quebec User Evaluation of Satisfaction with assistive Technology; SD: standard deviation; QOL: quality of life; SF-12: short form-12.

a Scores on a scale of 1–5 with 1 indicating *not satisfied at all* and 5 being *very satisfied*.

b Scores on a scale of 0–100 with higher scores indicating better quality of life.

## Reasons for withdrawal

The two main reasons for withdrawal from the pilot project were the change in RRT modality (n = 4) and death (n = 2). The three earliest dropouts were at day 8 (renal transplantation), day 18 (death) and day 86 (dislike of technology). Other reasons for discontinuation are illustrated in Table 4. The retention rate excluding medical reasons was 91%.

**Table 4.** Reasons for withdrawal from pilot project.

Reasons for withdrawal	Patients (n)	%
Medical reasons		
Renal transplant	3	13.6
Change to HD	1	4.5
Move to residential home	1	4.5
Died	2	9.1
Others		
Lost POD	1	4.5
Patient request	1	4.5
Total	9	40.9

HD: haemodialysis.

## Discussion

This is the first study in the United Kingdom that has utilised telehealth technology to promote self-management skills, assess user acceptability and collect data on admission avoidance in patients with ESRD on PD.

Previous reports from a Spanish group in stable patients undergoing treatment with PD demonstrated a reduction in the mean hospitalisation rate to 2.2 days/patient/year in the telehealth support group (n = 25 patients) in comparison to 5.7 days/patient/year in the control group (n = 32 patients). Time spent on teleconsultations was lower compared to hospital consultations (mean 22 versus 33 min, p < 0.01), and 89% (148 consultations) of the consultations lead to a medical intervention. In 96% cases (160 instances), patients felt telemedicine successfully replaced hospital visit. The study was conducted over a 2-year period in patients with a mean patient age of 48 ± 10 years.^9^ Our avoidance of admission data is similar. Interventions in our group occurred in 47% cases which is lower than the above report. This is possibly explained by the definition of intervention, as we did not record any intervention that was not directly related to an alert.

A recent systematic review of 14 studies to analyse the outcomes of active therapies with telemedicine in patients with stroke, medical oncology and nephrology concluded that telehealth for rural patients was promising with the need for further robust data to study effectiveness, feasibility and safety.^10^ Of the three studies on telehealth in dialysis, two^11,12^ were on HD patients. The third, on eight PD and three home HD patients, was a qualitative study of patient experiences and their views on how to optimise communication with health services and the role of telemedicine. Using semi-structured interviews, the authors recorded experiences based on imagination of what telemedicine could add to their daily care of home dialysis. Although the potential of telemedicine was hypothetical, patients especially those with machines felt telemedicine could create security and help choose a home-based treatment. There were further scopes to use this technology to improve communication and training. Some patients without machines (continuous ambulatory PD), however, did not see the added value.^13^

An earlier study of a telemedicine system for PD patients consisted of data collection from an APD machine, blood pressure monitor, weighing scale and an interview system via a digital camera and television. Data were collected on seven patients, one >90 years for 1–6 months (mean 3 months). The authors tentatively concluded that patients benefited from telemedicine and this improved QOL.^14^ This technology has since been further developed in PD patients and integrated with cellular telephone devices enabling data collection on a wide variety of parameters including exercise level and blood glucose monitoring.^15^

Our self-reported QUEST questionnaires demonstrate a positive experience of the use of telehealth technology, but this does not translate into overall improvement in QOL. Issues surrounding QOL are complex and often need to be addressed by more than a single intervention. This is beyond the remit of this study and needs further exploration.

The largest study evaluating cost-effectiveness of telehealth technology in the United Kingdom in patients with long-term conditions is the Whole System Demonstrator (WSD) trial. In this cluster randomised controlled study, 1573 participants with heart failure, COPD or diabetes were followed up for 12 months. The cost for the telehealth group was higher and the quality-adjusted life-years (QALYs) gained were similar to those receiving standard care. The results were calculated on self-reported questionnaires of service use and did not include surrogate clinical markers known to affect morbidity.^1^ This is in contrast to the 3-year study in high-risk dialysis patients in the United States with nurse oversight that showed cost savings by avoidance of hospitalisations and emergency room visits.^16^ Although we did not collect data on costs, the avoidance of 36 admissions supports the potential for significant savings. The differences in health set-ups across the continents, the study of different conditions and the methodologies in data collection may explain some of the differences seen.

## Limitations

Our study is limited by the small patient numbers, absence of a control arm and being unblinded. The small and decreasing number of patients on PD in the developed world, however, makes it difficult to perform a sufficiently powered study to detect a significant difference. It is possible that patients would have sought medical help through other means in the absence of telehealth technology, but it is likely to have taken longer.

Despite these limitations, we believe our data are robust. Admissions were prevented following assessment by nurse specialist on agreed standardised protocols (as previously described). Furthermore, some participants who required intensive monitoring were managed within the community supporting the prevented admission data further. The instantaneous nature of the alerts received by the clinical team adds to the strength of the telehealth approach.

Patients were not able to communicate with clinicians via PODs and did not receive notifications that alerts had been reviewed. The ability to hold such a two-way communication would be a valuable development.

## Conclusion

Telehealth is useful to monitor patients with ESRD on PD. It is acceptable across a wide age range and provides an additional resource for patients to self-manage their condition. Satisfaction scores and retention rates suggest a high level of acceptability. Further avenues in developing such technologies should be explored in renal patients.
